# Focused ultrasound for safe and effective release of brain tumor biomarkers into the peripheral circulation

**DOI:** 10.1371/journal.pone.0234182

**Published:** 2020-06-03

**Authors:** Lifei Zhu, Arash Nazeri, Christopher Pham Pacia, Yimei Yue, Hong Chen

**Affiliations:** 1 Department of Biomedical Engineering, Washington University in St. Louis, St. Louis, Missouri, United States of America; 2 Mallinckrodt Institute of Radiology, Washington University School of Medicine, St. Louis, Missouri, United States of America; 3 Department of Radiation Oncology, Washington University School of Medicine, St. Louis, Missouri, United States of America; George Washington University, UNITED STATES

## Abstract

The development of noninvasive approaches for brain tumor diagnosis and monitoring continues to be a major medical challenge. Although blood-based liquid biopsy has received considerable attention in various cancers, limited progress has been made for brain tumors, at least partly due to the hindrance of tumor biomarker release into the peripheral circulation by the blood-brain barrier. Focused ultrasound (FUS) combined with microbubbles induced BBB disruption has been established as a promising technique for noninvasive and localized brain drug delivery. Building on this established technique, we propose to develop FUS-enabled liquid biopsy technique (FUS-LBx) to enhance the release of brain tumor biomarkers (*e*.*g*., DNA, RNA, and proteins) into the circulation. The objective of this study was to demonstrate that FUS-LBx could sufficiently increase plasma levels of brain tumor biomarkers without causing hemorrhage in the brain. Mice with orthotopic implantation of enhanced green fluorescent protein (eGFP)-transfected murine glioma cells were treated using magnetic resonance (MR)-guided FUS system in the presence of systemically injected microbubbles at three peak negative pressure levels (0.59, 1.29, and 1.58 MPa). Plasma eGFP mRNA levels were quantified with the quantitative polymerase chain reaction (qPCR). Contrast-enhanced MR images were acquired before and after the FUS sonication. FUS at 0.59 MPa resulted in an increased plasma eGFP mRNA level, comparable to those at higher acoustic pressures (1.29 MPa and 1.58 MPa). Microhemorrhage density associated with FUS at 0.59 MPa was significantly lower than that at higher acoustic pressures and not significantly different from the control group. MRI analysis revealed that post-sonication intratumoral and peritumoral hyperenhancement had strong correlations with the level of FUS-induced biomarker release and the extent of hemorrhage. This study suggests that FUS-LBx could be a safe and effective brain-tumor biomarker release technique, and MRI could be used to develop image-guided FUS-LBx.

## Introduction

Central nervous system (CNS) tumors are significant causes of cancer morbidity and mortality, especially in children and young adults, where they account for ~20–30% of cancer deaths [1]. Noninvasive neuroimaging modalities, e.g., magnetic resonance imaging (MRI) and computerized tomography, have been used to evaluate tumor lesions, but observed changes, especially after treatment, can be difficult to interpret [2]. Surgical resection or stereotactic biopsy is typically performed for histologic confirmation and increasingly for genetic profiling. However, tissue biopsy requires brain surgery and can be associated with adverse effects such as hemorrhage and infection [3]. Repeated tumor biopsies that may be needed for tracking tumor evolution, treatment response, and tumor recurrence are often not feasible. Furthermore, tissue biopsies may be challenging when tumors are located at difficult locations, or patients are too ill to tolerate invasive procedures [4].

Noninvasive blood-based liquid biopsies are a rapidly emerging strategy to provide genetic tumor profiling that can be used for treatment selection, treatment monitoring, residual disease detection, and asymptomatic individual screening [5]. Some success has been achieved in integrating blood-based liquid biopsy into the routine clinical diagnostics [6]. However, limited progress has been made for brain tumors. Several groups have reported modest sensitivity for biomarker detection in the cerebrospinal fluid (CSF) of patients with brain tumors (positive in approximately 50% of patients) [7,8]. Apart from the limited sensitivity of CSF-based liquid biopsies, genetic mutations prevalent in the tumor tissue may diverge from those detected in CSF [9]. Blood-based liquid biopsies are noninvasive, but there remain multiple hurdles for their application in brain tumors. First, the blood-brain barrier (BBB) restricts the release of large molecules such as DNA and RNA from the tumor to the peripheral circulation [10]. Even though partial BBB disruption is a core feature of glioblastoma (the most common type of high-grade glioma) at the late stage of the disease, the BBB remains intact in the early-stage glioblastoma and in low-grade diffuse gliomas [11]. Second, brain tumors, especially glioblastomas, are heterogeneous with spatially distinct molecular profiling [12]. Localization of tumor areas that harbor specific mutations is not possible using conventional methods of liquid biopsy, as these methods are inherently spatially agnostic. Third, many tumor markers, such as some cell-free RNAs and DNAs, have short half-lives in blood [13]. Detection of these biomarkers can be enhanced by stimulating their release from the tumor to the circulation and precisely controlling the blood-collection time to be shorter than their lifetimes in the blood. This allows the release of tumor biomarkers that are concordant to the genetic make-up of the tumor. Therefore, a noninvasive, spatially resolved, and temporally controlled liquid biopsy technique is in great need to improve the clinical care of patients with CNS tumors.

Focused ultrasound (FUS) combined with microbubbles-induced BBB disruption technique (FUS-BBBD) has been established as a promising technique for noninvasive and localized brain drug delivery [14–16]. As a drug delivery technique, it has been explored in preclinical studies for the treatment of various neurological diseases [17,18], such as primary brain tumors [19–21], metastatic brain cancers [22], Alzheimer’s disease [23], and Parkinson’s disease [24]. On-going clinical trials are evaluating the feasibility and safety of this technique in patients with glioblastoma, Alzheimer’s disease, and amyotrophic lateral sclerosis [25–28]. Recently, we proposed that FUS-induced BBB disruption opens “two-way trafficking” between brain and blood, based on which we introduced the FUS-enabled brain tumor liquid biopsy technique (FUS-LBx) [29]. FUS-LBx offers a promising strategy that can provide noninvasive, spatially-resolved, and temporally-controlled brain tumor liquid biopsies. We demonstrated that FUS-LBx released tumor-specific biomarkers from brain tumors in murine glioblastoma models [29]. This approach is different from previously reported FUS-facilitated liquid biopsy techniques developed for *in vivo* biomarker release from cancers outside the brain [30–32]. In one of those reports, pulsed high-intensity focused ultrasound (HIFU) with high acoustic pressures (ultrasound frequency = 1.5 MHz, peak compressional focal pressure = 90 MPa, and peak rarefactional focal pressure = 17 MPa) was used to induce histotripsy (*i*.*e*., a technique for mechanical tissue fractionation) in a rat model of prostate cancer, and this enhanced the release of cell-free tumor microRNA into the blood circulation [30]. In another study, a chicken embryo tumor model was used to show the feasibility of amplifying the release of extracellular vesicles using HIFU (ultrasound frequency = 1.15 MHz and peak to peak pressure was within the range of 10–30 MPa) in combination with phase-change nanodroplets which changed to microbubbles upon HIFU sonication [31]. In a recent study, two protein biomarkers were found to be significantly increased in the plasma of patients undergoing HIFU thermal ablation (ultrasound frequency = 1.1 MHz and power of 100–200 W) of uterine fibroids [32]. All these previous studies used HIFU to induce permanent mechanical or thermal disruption of the tumor to enhance the release of tumor biomarkers. This tissue-damaging effect of HIFU limits the application of these techniques as diagnostic tools in a sensitive organ such as the brain. In contrast, the FUS-LBx technique we proposed uses low-intensity pulsed FUS, which has the potential advantage of enabling biomarker release without causing tissue damage. However, in our previous proof-of-concept study [29], the acoustic pressures used were intentionally selected to be relatively high (1.52–3.53 MPa) to increase the chance of success in releasing biomarkers. As expected, hemorrhage was found in the FUS-targeted tumor area in that study.

The objective of the current study was to demonstrate that FUS-LBx could sufficiently increase plasma levels of the tumor biomarkers without causing hemorrhage in the brain. We performed FUS-LBx with different acoustic pressures and quantified the level of induced tumor biomarker release and the extent of associated hemorrhage. We further explored whether post-sonication changes in tumor MR contrast enhancement could predict successful biomarker release for the future development of image-guided FUS-LBx technique.

## Results

### Biomarker release level quantification

**Fig 1:**
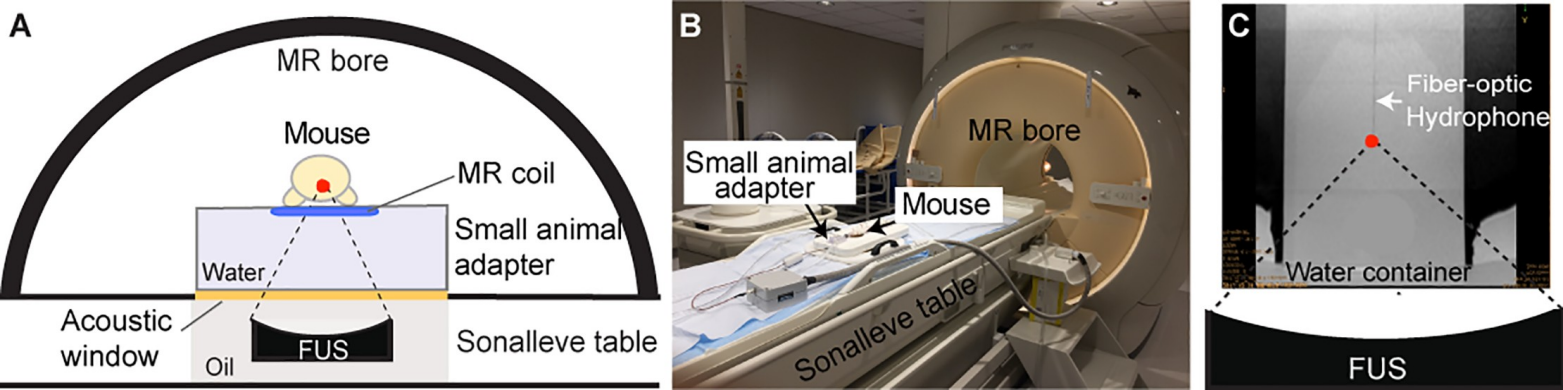
Experimental setup. (A) Illustration and (B) picture of the MR-guided FUS system used for the FUS-LBx treatment. A clinical MR-guided FUS system that integrated a clinical MRI scanner with a FUS phased array was configured for small animal study by coupling with a small animal adaptor (see Methods for more details). (C) MR image of a fiber-optic hydrophone used for measuring the acoustic pressures. The hydrophone was placed in a water container that was placed on top of the small animal adapter.

Mice with orthotopic implantation of enhanced green fluorescent protein (eGFP)-transfected murine glioblastoma cells were recruited into this study when the maximum diameter of the tumor reached 2 mm. The mice were treated using an MR-guided FUS system (Fig 1A and 1B) at three different peak negative acoustic pressure (PNP) levels: (i) 0.59 MPa; (ii) 1.29 MPa; (iii) 1.58 MPa (n = 5 in each group). The acoustic pressure output of the FUS transducer was calibrated using a fiber-optic hydrophone following our previously published method (Fig 1C) [33,34]. eGFP mRNA was selected as a representative tumor-specific nucleic acid biomarker because it is highly specific to our tumor model, mRNA is more abundant in the plasma than the more intensively investigated ctDNA [35], and mRNA can be detected using established polymerase chain reaction (PCR)-based approaches [36], which provides a reliable benchmark test to evaluate the performance of FUS-LBx.

**Fig 2:**
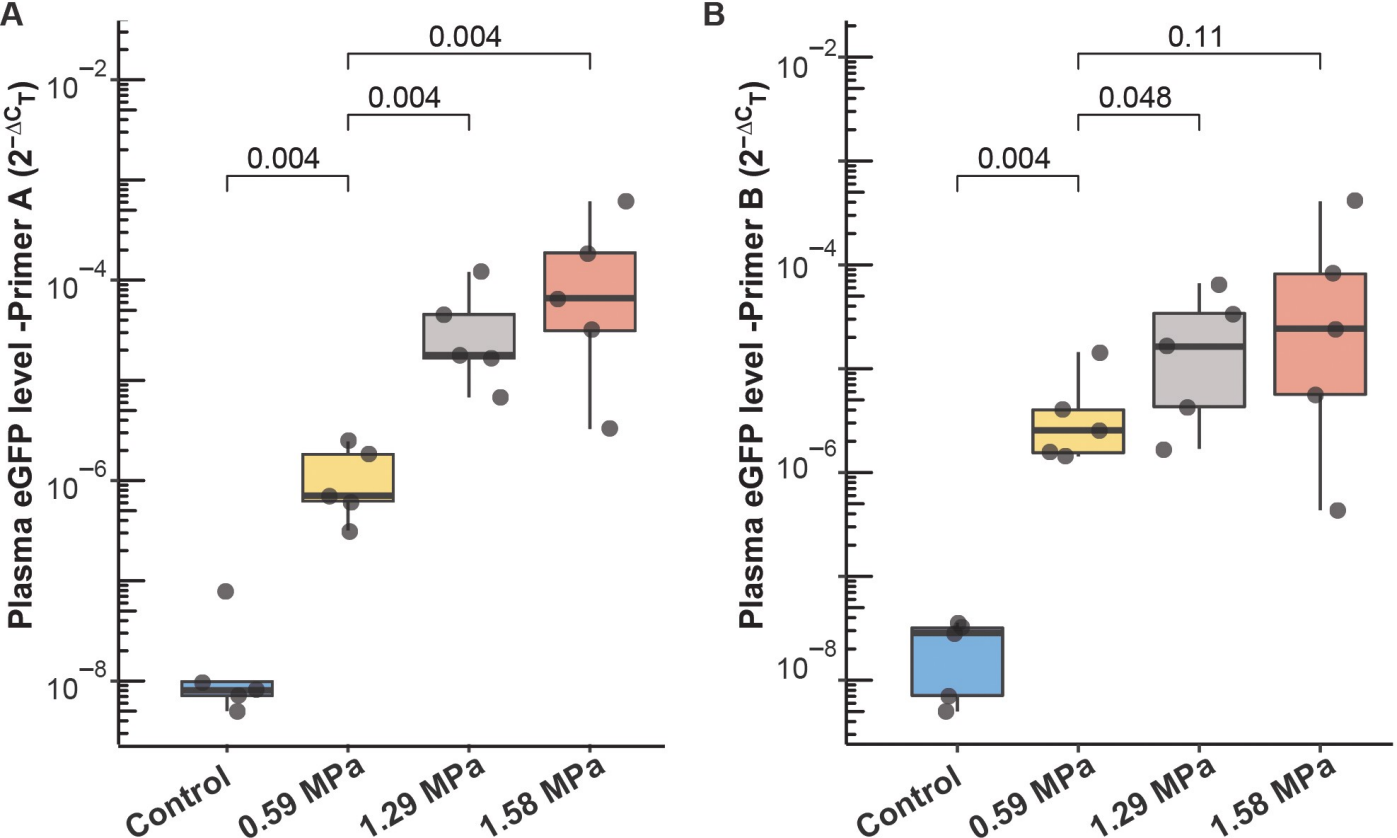
Effect of peak negative pressure on FUS-LBx yield. Comparison of the circulating eGFP mRNA level in the control mice receiving no treatment and FUS-treated mice at 0.59, 1.29, and 1.58 MPa. eGFP mRNA levels were quantified using quantitative polymerase chain reaction (qPCR) with (A) primer A and (B) primer B.

Two primer sets were used to improve the robustness of the quantitative PCR (qPCR) analysis. When compared to the control group (no FUS and microbubble), all three FUS-treated groups demonstrated significant increases in plasma eGFP mRNA levels (all FUS-treated mice vs. control group: one-tailed *p =* 6.5 × 10^−5^ for both primer sets: primers A and B; Table 1, Fig 2). When compared with the control group, there were 55-fold (Primer A: one-tailed *p =* 0.004) and 221-fold (Primer B: one-tailed *p =* 0.004) increase in plasma eGFP mRNA levels in mice that received FUS with the lowest PNP (0.59 MPa). Plasma eGFP mRNA levels in mice sonicated by FUS of 1.29 MPa and 1.58 MPa achieved respectively about 2,000-fold and 8,000-fold average enhancement relative to the control group, respectively (Table 1). For primer A, plasma eGFP mRNA levels were significantly greater with high acoustic pressures when compared to 0.59 MPa (1.29 MPa vs. 0.59 MPa: 35-fold higher, one-tailed *p =* 0.004; 1.58 MPa vs. 0.59 MPa: 151-folder higher, one-tailed *p =* 0.004). These effects were less pronounced when eGFP mRNA levels were measured using primer B (1.29 MPa vs. 0.59 MPa: 5-fold higher, one-tailed *p =* 0.048; 1.58 MPa vs. 0.59 MPa: 22-fold higher, one-tailed *p =* 0.11).

**Table 1:** Group average ± standard deviation of eGFP RNA levels, hemorrhage densities, and tumor MRI enhancement.

Group	Control	0.59 MPa	1.29 MPa	1.58 MPa
**eGFP mRNA level (Primer A), 2**^**-ΔCT**^	2.15×10^−8^±3.13×10^−8^	1.19×10^−6^±9.10×10^−7^	4.15×10^−5^±4.66×10^−5^	1.80×10^−4^±2.51×10^−4^
**eGFP mRNA level (Primer B), 2**^**-ΔCT**^	2.17×10^−8^±1.45×10^−8^	4.79×10^−6^±5.47× 10^−6^	2.45×10^−5^±2.66×10^−5^	1.04×10^−4^±1.73×10^−4^
**Hemorrhage density (%)**	0.12±0.06	0.17±0.08	0.95±0.58	1.69±1.05
**Tumor MR enhancement**	12.48 ± 4.05	13.68±3.91	13.51±3.58	13.69±2.20

### Tissue hemorrhage quantification

**Fig 3:**
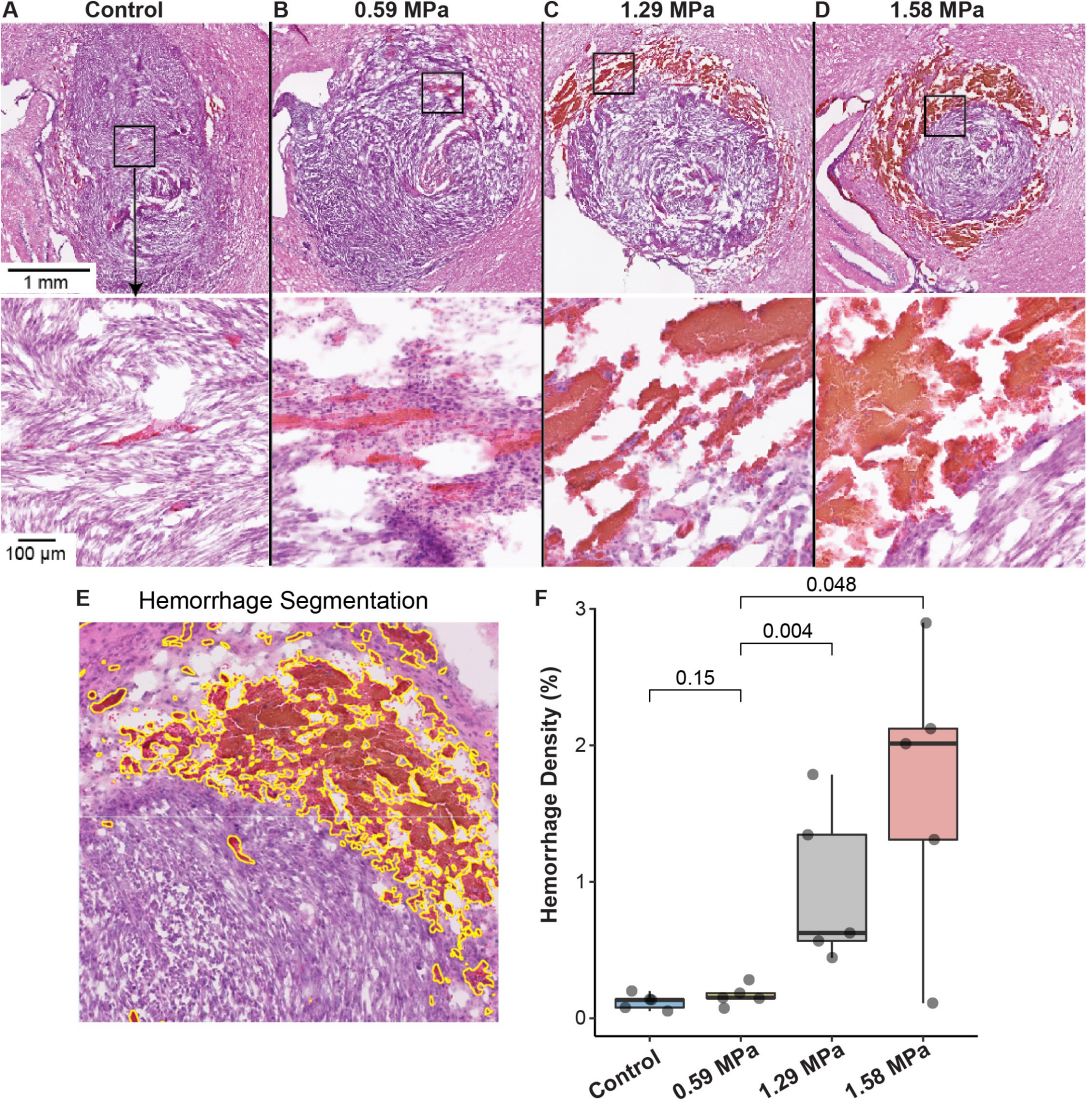
Effect of peak negative pressure on brain hemorrhage. (A–D) Representative images of H&E-stained sections from mice in the control, 0.59 MPa, 1.29 MPa, and 1.58 MPa groups, respectively. Boxes in the images on the top indicate the location where the higher magnification images (bottom) were acquired. (E) Illustration of microhemorrhage quantification using the positive pixel detection algorithm (see Methods). Yellow lines highlight the detected microhemorrhage. (F) Microhemorrhage density is significantly higher in mice treated with higher pressures (1.29 MPa and 1.58 MPa) compared to the low pressure (0.59 MPa) and control groups.

Hematoxylin and eosin (H&E) stained sections of the brain were examined for the presence of microhemorrhages. Representative images of the H&E-stained sections are shown in Fig 3A–3D. Microhemorrhage was most evident in the periphery of the tumor in mice treated with higher PNPs. After color deconvolution, areas of microhemorrhage were identified using the positive-pixel count algorithm (Fig 3E). Microhemorrhage density was calculated as the proportion of the surface area of microhemorrhage to the total surface area of the evaluated brain tissue in a given slice. There was no significant difference in microhemorrhage density between mice treated with 0.59 MPa FUS and the control mice (Table 1, Fig 3F). The microhemorrhage density was significantly higher in 1.29 MPa and 1.58 MPa groups than the 0.59 MPa and control groups. Microhemorrhages seen in the control mice were predominantly scattered in the tumor. In addition to scattered intratumoral microhemorrhage, peritumoral hemorrhage near the interface of the tumor and normal brain parenchyma was seen in 1 out of 5 mice treated with 0.59 MPa FUS as well as in 4 out of 5 mice in the 1.29 MPa and 1.58 MPa FUS-treated groups. Large variations in the hemorrhage density were observed within each group, especially at higher pressures (1.29 MPa and 1.58 MPa). No off-target damage to the surrounding brain tissue was seen in any examined brain image (see S1 Fig).

### Predicting biomarker release level and hemorrhage using MRI

**Fig 4:**
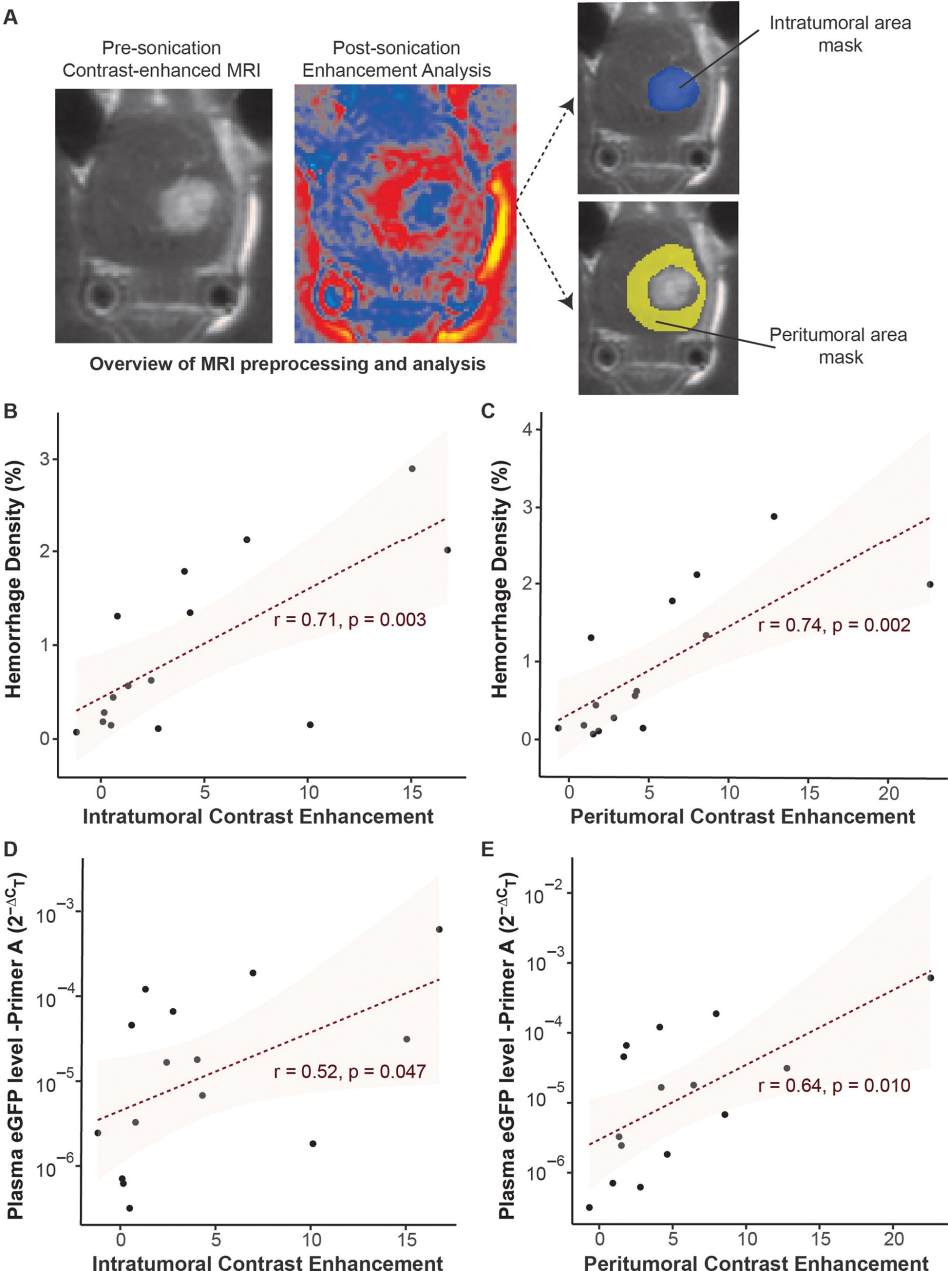
Relationships between intratumoral and peritumoral MR image contrast enhancements and microhemorrhage density and eGFP mRNA level. (A) Overview of MRI analysis workflow. A representative pre-sonication MR image is presented to show the tumor. This image was used to define the intratumoral area mask and peritumoral area mask. Pre- and post-sonication contrast-enhanced MR images were processed following the same procedure described in Method for the quantification of MR contrast enhancement in the intratumoral area and the peritumoral area. Correlation analyses between intratumoral and peritumoral enhancements with microhemorrhage density (B, C) and plasma eGFP mRNA level (D, E) in FUS-treated mice (n = 15) using primer A.

Increased MRI image intensity on contrast-enhanced MR images was seen both within the tumor and in the peritumoral area of the tumor after sonication (Fig 4A). Since there is a significant difference between intratumoral and peritumoral enhancement before sonication, we opted to evaluate post-sonication intratumoral and peritumoral enhancement separately. We interrogated possible associations between post-sonication enhancements and microhemorrhage density and FUS-LBx yield. Greater sonication-induced intratumoral and peritumoral contrast enhancement were associated with higher hemorrhage burden (Fig 4B and 4C) and greater post-sonication plasma eGFP mRNA levels detected using primer A (Fig 4D and 4E). The correlation coefficients were lower for eGFP levels (*r* = 0.52 for intratumoral contrast enhancement and *r* = 0.64 for peritumoral enhancement) than hemorrhage burden (*r* = 0.71 for intratumoral contrast enhancement and *r* = 0.74 for peritumoral enhancement). Similar associations were seen between intratumoral/peritumoral enhancements and eGFP mRNA levels measured with primer B (*r* = 0.51 for intratumoral contrast enhancement and *r* = 0.56 for peritumoral enhancement). The correlation coefficients for all the above analysis were slightly higher with peritumoral contrast enhancement than intratumoral contrast enhancement.

## Discussion

We demonstrated that the FUS-LBx technique could be applied with low acoustic pressure without significant sonication-induced hemorrhage using a murine orthotopic glioblastoma model. MRI analysis revealed that post-sonication intratumoral and peritumoral hyperenhancement were associated with the level of FUS-induced biomarker release and the extent of hemorrhage.

Our study showed that FUS-induced release of eGFP mRNA from the tumor into the blood circulation is higher when FUS with high PNPs (1.29 and 1.58 MPa) was applied compared with relatively low pressure (0.59 MPa) (Fig 2). The reason for this difference might be that higher PNPs may lead to an increased likelihood of microbubbles undergoing violent inertial cavitation that can, in turn, induce greater disruption of the BBB [37–40]. In this way, a higher amount of eGFP mRNA may be released to the bloodstream. In our previous study, we found the eGFP mRNA level was not pressure-dependent within the range of 1.52–3.53 MPa. All these findings combined suggest that as FUS pressure increases, the biomarker release level increases and then reaches a plateau. This may be in part due to the potentially paradoxical effects of increasing PNP on the FUS-LBx yield. On the one hand, higher PNP can result in more significant BBB disruption. On the other hand, it can result in vascular damage and decreased tumor perfusion, and hinder tumor biomarker release into the peripheral circulation.

For the evaluation of the brain hemorrhage, our results showed a significantly higher microhemorrhage when comparing the 1.29 MPa and 1.58 MPa groups with the 0.59 MPa group or the control group (Fig 3). Although there was no statistically significant difference in the extent of microhemorrhage between the control group and the FUS-sonicated group at 0.59 MPa, we observed microhemorrhage beyond the bounds of the tumor along the interface between the tumor and normal brain tissue in one out of five cases. This pattern of hemorrhage was more pronounced with higher PNPs (Fig 3C and 3D). This pattern of hemorrhage in the tumor periphery may result from the immaturity and instability of peritumoral vasculature rendering them more susceptible to mechanical injury induced by FUS.

Relatively large variations within each group were observed in the analysis of both FUS-LBx yield and FUS-induced hemorrhage. Such variations among repeated FUS treatments have been reported before in FUS-induced BBB disruption experiments [41–44]. These variations can be caused by changes in parameters that are hard to control, such as the size distribution of microbubbles that reach the targeted brain location [41], circulating microbubble concentration in blood [42], blood vessel density within the treated region of the tumor [43], and heterogeneous acoustic property of skull [44]. Treatment monitoring techniques, such as passive cavitation detection/imaging [45–47], are needed to directly detect and quantify microbubble cavitation behavior and characterize variations among repeated FUS treatment. Future work is needed to integrate FUS-LBx with passive cavitation detection/imaging techniques to evaluate whether we can find correlations between the cavitation level/dose [16,45,48] and the level of tumor biomarker release as well as the extent of associated hemorrhage. If these relationships can be defined, feedback control algorithms can be implemented based on cavitation detection/imaging to control the FUS output during the treatment to minimize variations associated with the FUS treatment and improve the consistency.

Our study also presented that greater contrast enhancement was associated with both a greater level of eGFP mRNA plasma level and a higher hemorrhage density (Fig 4). Higher post-sonication intratumoral and peritumoral contrast enhancements were both associated with more elevated tumor markers in plasma. At the same time, post-sonication enhancement within the tumor and in its periphery were associated with microhemorrhage density. MR imaging markers that can predict safe and successful liquid biopsy are essential for translation of FUS-LBx to the clinic, as they can inform the clinician for sufficiency of FUS application for liquid biopsy or need for a higher magnitude of PNP. Future studies are needed to explore the potential of MRI in the assessment of FUS-LBx outcome and safety. MRI can be combined with passive cavitation imaging to develop image-guided FUS-LBx, which employs real-time passive cavitation imaging for FUS-LBx treatment monitoring and feedback control and utilizes MRI for treatment outcome and safety assessment.

The FUS-LBx technique is built on the established FUS-BBBD technique. FUS-BBBD has been developed for delivering therapeutic agents from the blood into the brain parenchyma. FUS-LBx is proposed for releasing brain tumor-specific biomarkers from the brain into the blood. Both techniques utilize FUS in combination with microbubbles to disrupt the BBB. FUS-BBBD studies in rodents typically use FUS with acoustic pressure around 0.5–0.7 MPa at a center frequency of around 1 MHz for brain drug delivery [49,50]. The current study found, for the first time, that FUS-LBx achieved successful brain tumor biomarker release at an acoustic pressure as low as 0.59 MPa (center frequency of the FUS was 1.44 MHz), which was comparable to the acoustic pressures commonly used for FUS-BBBD [21,24,51]. Short- and long-term safety of FUS sonication at similar pressure levels have been demonstrated in small animal [52,53] and large animal studies [52,54,55] using histological analysis, functional neuroimaging, electrophysiological measurements, and behavior testing [56]. Our finding is significant because it demonstrated that FUS-LBx could be a safe and effective technique for brain tumor biomarker release. Moreover, the safety of FUS-induced BBBD has been demonstrated in several clinical studies [26,28,57,58]. Previous clinical studies using a 1 MHz ultrasound device that was implanted in the skull bone found that the BBB was disrupted at acoustic pressure levels up to 1.1 MPa without detectable damage [28,57]. The 0.59 MPa at a comparable ultrasound frequency of 1.44 MHz is considered within the safe pressure levels identified in those clinical studies. Experience and knowledge gained from these clinical studies would provide valuable guidance for the future clinical translation of the FUS-LBx technique.

There are limitations of our study that need to be considered. First, we used plasma mRNA levels of eGFP that was uniquely expressed in the tumor cells to evaluate FUS-mediated liquid biopsy. While this approach is adequate to determine the effectiveness of FUS-LBx, it may not fully recapitulate the clinical settings for liquid biopsy. Since human glioblastoma does not have the intrinsic eGFP expression, the efficacy of FUS-LBx in detecting naturally occurring tumor-specific biomarkers [59] needs to be determined. Second, similar to our prior study [29], we collected blood using terminal cardiac puncture as mice have a small total blood volume (~1.5 mL). Future study could be performed by collecting blood samples at different time points in different groups of mice to evaluate the temporal course of FUS-mediated biomarker release. Future studies could also be performed using large animal models, such as pigs and non-human primates, for serial blood collection from the same animal. Fundamental differences between humans and rodents in skull and brain anatomy will require a comprehensive evaluation of the FUS-LBx technique in large animal models before the clinical translation. Third, we observed a few inconsistencies in plasma eGFP mRNA findings with different primer sets. Although eGFP mRNA measured with both primer sets were overall highly correlated (*r* = 0.93, root mean square difference = 1.85 cycle threshold), the difference between plasma eGFP mRNA levels of mice treated at 0.59 MPa and higher PNPs were less prominent with primer set B. More robust and reproducible measurements of RNA levels with droplet digital PCR [60] may mitigate these issues and enable reliable detection of small differences in FUS-LBx. Finally, future studies are warranted to evaluate the effects of the tumor type, size, location, and stage on the biomarker release efficiency by FUS-LBx. These tumor-related parameters are essential as they can control the tumor vessel size, density, and permeability, which are expected to influence the microbubble behaviors and the biomarker release efficiency.

## Conclusions

Our study showed that acoustic pressure as low as 0.59 MPa was sufficient for FUS-LBx. Although higher peak negative acoustic pressures tended to be associated with a better yield of liquid biopsy, it was also associated with a higher burden of microhemorrhage. MRI can be used in not only guiding the FUS targeting of a specific brain region but also providing predictions of the biomarker release level and potential hemorrhage extent. Future studies are needed to develop MRI-guided FUS-LBx to improve its safety and efficacy.

## Methods and methods

### FUS-LBx treatment procedure

Animal housing, husbandry, and procedures were reviewed and approved by the Institutional Animal Care and Use Committee of Washington University in St. Louis in accordance with the National Institutes of Health Office of Laboratory Animal Welfare (protocol #: 20180185). Mice (NIH Swiss mice, Strain 550, 6–8 weeks, female, n = 20, Charles River Laboratory, Wilmington, MA, USA) were used in this study. Animals were fed *ad libitum* with standard laboratory chow and water in cages located in a room under a 12-hour light/dark period (light on 8:00 AM) at controlled temperature (22–24°C) and humidity (30%–70%). All procedures were performed under general anesthesia sustained using isoflurane at a concentration of 1–2.5% and all efforts were made to minimize suffering.

Mouse glioma GL261 cell lines were obtained from Dr. Dinesh Thotala (Washington University School of Medicine, St. Louis, MO, USA) and cultured in Dulbecco's Modified Eagle Medium (DMEM) with Nutrient Mixture F-12 1:1, 10% fetal bovine serum, 1% sodium pyruvate (Life Technologies, USA) in a 5% CO_2_ incubator at 37°C. The absence of mycoplasma in the culture was confirmed by using the MycoAlertTM Mycoplasma Detection Kit (Lonza, Basel, Switzerland). Mice were anesthetized and fixed into a stereotactic head frame. A paramedian incision was made on the scalp, and a 1-mm burr hole was drilled 2 mm posterior and 1.5 mm lateral to the bregma. eGFP-transduced GL261 (volume: 5 μL; cell count: about 50,000 cells) were mixed with CorningTM Matrigel (Catalog 356231, Corning Life Science, New York, USA) and injected through the burr hole using a syringe. The burr hole was sealed with bone wax, and the skin incision was glued together with tissue glue. We observed the animals’ behavior and attended any difficulties the animals might experience at least once per day. Post-surgery analgesia was provided by subcutaneous injection of buprenorphine (0.03 mg/kg in saline twice daily) for 3 days.

The growth of the tumor was monitored using a clinical MRI scanner (Ingenia 1.5T, Philips Healthcare, Best, The Netherlands) coupled with a small animal coil (FUS Instruments Inc., Toronto, Ontario, Canada) as shown in Fig 1 twice per week. Once the maximum diameter of the tumor reached 2 mm (at around post-implantation days 15) as measured based on the contrast-enhanced MRI, the mice were randomly assigned into four groups: a control group that received no treatment (n = 5) and three groups that received FUS treatment at three different pressure levels with n = 5 for each level. Based on the contrast-enhanced MR images, there were no significant differences in the tumor volume among all the groups before FUS sonication. All other parameters were the same across the treatment groups: FUS center frequency = 1.44 MHz, sonication duration = 240 s, treatment target = 1 (at the center of the tumor), pulse repetition frequency = 1 Hz, duty cycle = 1%, and pulse length = 10 ms.

The details of the sonication procedure and the dose and concentration of the microbubbles have been described previously[29]. Briefly, mice were treated by FUS using a clinical MR-guided FUS system (Sonalleve V2, Profound Medical Inc., Mississauga, Canada) that integrated a clinical MRI scanner (Ingenia 1.5T, Philips Healthcare, Best, the Netherlands) with a 256-element phased-array FUS transducer. The system was configured for small animal study by coupling with a small animal adaptor (FUS Instruments Inc., Toronto, Ontario, Canada) placed above the acoustic window of the FUS transducer (Fig 1A and 1B). The adaptor consisted of a small animal coil, a standoff, and a mouse bed. The software of the MR-guided FUS system was modified to achieve tumor targeting under the guidance of MR images of mouse brains acquired using the small animal coil. It was also modified to perform pulsed FUS sonication at low-pressure levels used in this study by only turning on 128 elements. The output of the FUS transducer was controlled by setting the nominal acoustic power to be 10 W, 20 W, or 30 W. The acoustic fields generated by the FUS transducer was calibrated by a fiber optic hydrophone using our previously published methods (Fig 1C)[33,34]. The measured PNPs for the three acoustic power settings were 0.59 MPa, 1.29 MPa, and 1.58 MPa, respectively. The full width at half maximum of the axial beam and lateral beam was 12.10 mm and 1.37 mm, respectively.

Contrast-enhanced T1-weighted turbo spin-echo MR images (TR, 500 ms; TE, 13 ms; acquisition matrix, 96 × 96; resolution, 0.2 mm × 0.2 mm × 0.5 mm) were acquired before and after the FUS treatment to quantify changes in intratumoral and peritumoral enhancement.

### Plasma eGFP mRNA level quantification

Blood samples of 500–800 μL were collected from the heart about 20 min after FUS sonication and prepared for qPCR analysis of eGFP mRNA. The methods of qPCR analysis of eGFP mRNA have been described in our previous publication[29]. Briefly, RNA was extracted from the plasma samples using miRNeasy serum/plasma kit (Catalog no. 217184, Qiagen, USA) followed by Agencourt RNAClean XP beads (Catalog no. A63987, Beckman Coulter Inc., USA). Extracted RNA was then converted to cDNA using the Applied Biosystems high-capacity cDNA reverse transcription kit (Catalog no. 4368814, Thermo Fisher Scientific, USA). Two primer sets were used to quantify eGFP levels. We used 5.8S rRNA as the internal control to normalize the PCR data by calculating cycle threshold change (ΔC_T_) by subtracting C_T_ of the eGFP (C_T,eGFP_) by the C_T_ of the housekeeping gene, 5.8s rRNA (C_T,5.8S_). The relative gene expression level was determined using the 2^-ΔCT^ method: $2^{-\Delta C_{T}}=2^{-(C_{T,eGFP}-C_{T,5.8s})}$.

### MR image analysis

MR image processing and analysis were performed using tools available in FSL (FMRIB's Software Library) v5.0.10^36^. First, tumor regions were segmented semi-automatically on the pre-sonication contrast-enhanced T1-weighted images using ITK-SNAP v3.6.0^35^. Second, on each pre-sonication contrast-enhanced T1-weighted MR image of the brain tumor, a spherical control mask with 1 mm radius was drawn in the normal-appearing brain, and its mean and standard deviation were calculated. Third, all contrast-enhanced T1-weighted images (both pre-sonication and post-sonication images) were intensity normalized by subtracting the mean and then dividing by the standard deviation of the signal intensity within the control mask. Fourth, the peritumoral area was defined as the brain regions within 2 mm vicinity of the tumor. A preliminary mask was created using the *distancemap* command in FSL. This mask was then edited manually to exclude voxels outside the brain parenchyma. Finally, to quantify post-sonication changes in MRI contrast enhancement, the difference between normalized post-sonication and pre-sonication T1-weighted images was calculated within the intratumoral and peritumoral area masks.

### Histologic analysis

After blood collection, all mice were transcardially perfused with 0.01 M phosphate-buffered saline (PBS) followed by 4% paraformaldehyde under general anesthesia. Brains were harvested and prepared for paraffin sectioning. The mouse brains were horizontally sectioned to 15 μm slices and used for H&E staining. Images of the H&E-stained tissue sections were digitally acquired using Nanozoomer 2.0-HT slide scanner (Hamamatsu Photonics, Hamamatsu City, Japan). For each mouse, the section with the largest tumor surface area and least artifact was selected for quantitative analysis. QuPath v0.1.3 [61] was used to detect areas of microhemorrhage. After color deconvolution (hematoxylin vs. eosin), areas of microhemorrhage was detected using the positive-pixel count algorithm. Microhemorrhage density was calculated as the percentage of microhemorrhage surface area over the entire evaluated surface area (%).

### Statistical analysis

All statistical analyses were performed using R statistical software v3.5.0 (https://www.R-project.org/) and graphically displayed using *ggplot2* package v2.2.1. For group comparisons, the non-parametric Mann–Whitney U test was used. Associations between two continuous variables were assessed using Pearson correlation analysis. eGFP plasma levels were log transformed for correlation analysis. Statistical significance was set at *p* < 0.05.

## Supporting information

S1 Fig Representative H&E staining of whole brain slices obtained from the control group (A) and 0.59 MPa FUS-treated group (B). FUS was targeted at the tumor. No off-target damage to the surrounding brain tissue was seen in any examined brain images.
(DOCX)
