# Supplementary material for: Focused ultrasound for safe and effective release of brain tumor biomarkers into the peripheral circulation
Source: PLoS One. 2020 Jun 3;15(6):e0234182. doi: 10.1371/journal.pone.0234182 (PMC7269259; doi:10.1371/journal.pone.0234182)
Supplement: S1 Fig — Representative H&E staining of whole brain slices obtained from the control group (A) and 0.59 MPa FUS-treated group (B). FUS was targeted at the tumor. No off-target damage to the surrounding brain tissue was seen in any examined brain images. (DOCX) [file pone.0234182.s001.docx]

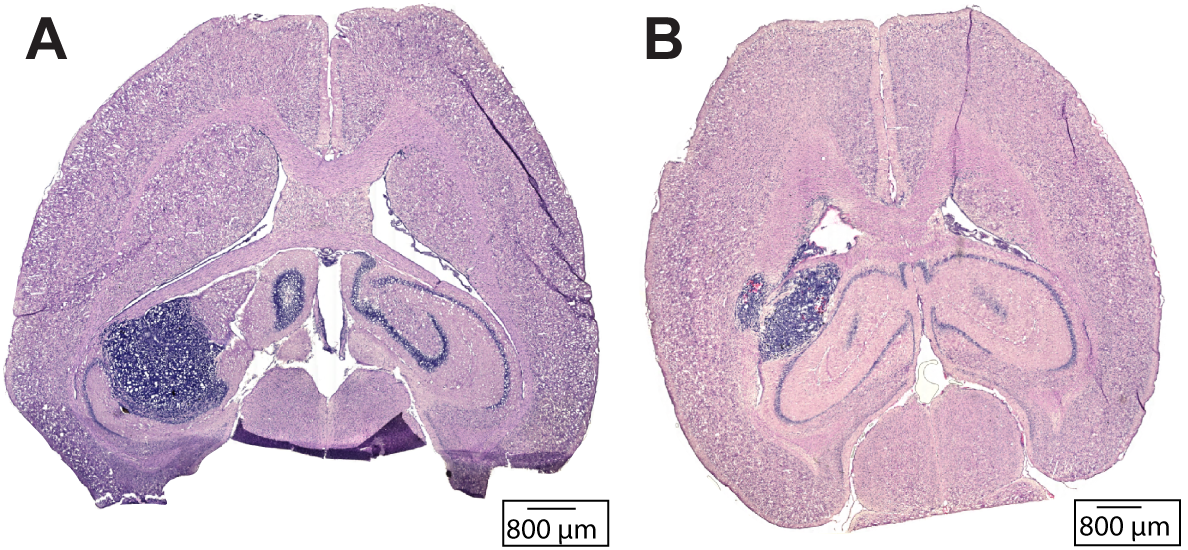


**S1 Fig.** Representative H&E staining of whole brain slices obtained from the control group (A) and 0.59 MPa FUS-treated group (B). FUS was targeted at the tumor. No off-target damage to the surrounding brain tissue was seen in any examined brain images.
